# The life cycle of a genome project: perspectives and guidelines inspired by insect genome projects

**DOI:** 10.12688/f1000research.7559.1

**Published:** 2016-01-05

**Authors:** Alexie Papanicolaou

**Affiliations:** 1 Hawkesbury Institute for the Environment, University of Western Sydney, Richmond, NSW 2753, Australia

**Keywords:** Genome sequencing, Bioinformatics education, opinion in bioinformatics, insect genomics, biocuration

## Abstract

Many research programs on non-model species biology have been empowered by genomics. In turn, genomics is underpinned by a reference sequence and ancillary information created by so-called “genome projects”. The most reliable genome projects are the ones created as part of an active research program and designed to address specific questions but their life extends past publication. In this opinion paper I outline four key insights that have facilitated maintaining genomic communities: the key role of computational capability, the iterative process of building genomic resources, the value of community participation and the importance of manual curation. Taken together, these ideas can and do ensure the longevity of genome projects and the growing non-model species community can use them to focus a discussion with regards to its future genomic infrastructure.

## Introduction

In this perspectives and opinion article, created from the viewpoint and experience of an insect genome informatician, I seek to explain how the generation, maintenance and publication of genome projects has now reached a stage that requires the community to pause for thought. Each genomics community is at a crossroads while it decides on what is the best strategy for generating and using high quality genome projects. There are undoubtedly leaders who have been pursuing specific strategies but the community is not necessarily on the same path. For over a decade, dozens of genome projects have been completed and this number is increasing exponentially. Due to my experience around insect genomics and participation in the i5k activities (an international consortium providing leadership and resources for insect genome projects ^1^), I shall present my views centred around the insect community but hopefully these views are broadly applicable. Previously, a co-author and I explained how the new, cheaper technologies changed the landscape by which genome projects are conceived and organised in the hope we can guide a burgeoning insect genomics community ^2^. We also argued that individual researchers can produce a genome reference sequence for their favourite species and the large consortia are no longer needed. Since then, we have seen first-hand how an incredible worldwide effort has managed to shed light on why and how we can initiate hundreds (if not thousands) of insect genome projects ^1^. The i5k’s aim is to educate and support individual scientists as they seek to acquire genomics skills. It has also produced the first tranche of key insect genomes, mainly picked due to their place in the phylogenetic tree, an achievement which may be currently underutilised but whose importance cannot be understated. After involvement with multiple genome projects, a diverse array of transcriptomic projects and at least one widely used genomics software, I have come to conclude that in our rush to initiate the genomes projects of a larger part the tree of life we have neglected some important issues. First, the only reason our throughput is so high is because all published genome papers present merely just a draft. It is a useful draft but still only an initial effort. Second, this draft does indeed contain many of the instructions of how to generate an organism, but a genome sequence alone does not decipher it. It merely transcribes so we can conduct experiments with it. Deciphering will require both good experimental design and the capability to integrate such experiments. Third, the research community is not just the end-user but also part of the project team; we have, on the whole, neglected to bring them up to speed. These issues may seem intuitive but much of the community’s leadership is not conscious of it. Such issues cannot be readily resolved without substantial education. In this opinion article, I present some of the insights I gained from my own journey that I believe can help inspire the community to follow us as we hack our way through the multi-species genome sequencing trail.

## Insight 1: A brave new, informatics-led, capability

During the last few years, genomics has moved from being a capability led by – and limited by – wet lab techniques to one led by computational science. One of the first eukaryotic genomes ever assembled was *Drosophila melanogaster* ^3^, chosen partly because its genome architecture is much simpler than that of the human one. At the time, there was much discussion on whether sheer computational power would have been able to solve such a puzzle. The history of how this affected the human genome project is well known. There were two camps with many inspiring individuals in each. One side was the publicly-funded effort to sequence the human genome who believed that ‘clone-at-a-time’, overlap-based sequencing was the answer. One the major forces of innovation from that group was Eric Lander. Originally a mathematician, his team at the MIT was responsible for creating a completely automated sequencing pipeline ^4^. As the pressure to publish was looming, the team realised that they did not have the computational software to generate the final genome assembly (until the skill and leadership of Jim Kent saved the day). The privately-funded side (TIGR and Celera), was led by a computational approach from the onset. Eugene Myers - having already worked on efficient computational approaches for biology (e.g. BLAST, suffix arrays and assembly) - proved that new computer science algorithms had the power to solve this problem much more efficiently ^5^. Their computational capability drove the development for a new wet-lab technique called Whole Genome Shotgun (WGS) sequencing. The first camp, in other words, built computational approaches to match the wet lab technique. The second one built a wet-lab capability to match a novel computational approach. The majority of the molecular biologists at the time did not support this WGS approach as it was rightly considered to be of inferior quality. At the end of the day, however, it was the cross-talk of the two capabilities that not only produced a comprehensive human genome but also allowed many other species to have their genome sequencing completed. Indeed, the human genome project completely changed our perception of how biological research can scale in a world that transcends borders and how it should maintain its science-led focus (see 6). Even though we still follow most of these tenets, as shown from the vast majority of genome project publications, genome sequencing is an enterprise inherently focused on building resources. That does not mean that it is no longer science-led, it is just the science is not always the fields of biochemistry, ecology or genetics.

For the human genome project, computer science approaches were used to not only convert the wet-lab data to something the research community could make use of but to also guide the wet-lab techniques. By now, an entire coterie of associated industries such as information technologies (IT), informatics (the use of IT to handle large datasets) and data science is underpinning much of genomics. These sciences, often lumped into the rather over-used but useful umbrella definition of ‘bioinformatics’, have not only allowed us generate genomes but have now become an integral part of any downstream experiment using a reference sequence. Generally, the wider community is not aware of the true potential of informatics as it could be. The benefits go beyond being able to analyse a dataset: The epistemological understanding that comes with studying statistics and informatics can provide the skills for integrating the multi-disciplinary and ever-increasing amounts of data and the framework to make sense of a more synthesized knowledge. If we – as educators - allowed for aspects of programming and data science to become an integral part of the undergraduate curriculum - rather than the lip service that is currently common in most institutions – then we not only equip the next generation with a set of skills but we inspire a uniquely effective way of dissecting complex problems. Further, the high-throughput analysis of data is now a core requirement for any genomic experiment, yet often the analysis is delegated to computer programs (bioinformatic software), which are, effectively, “black boxes”. Such software are great for enhancing productivity but they ought not to be used before we understand all assumptions made on our behalf and explore their parameter space for each particular dataset ^7^. Perhaps a way to resolve this is for the software engineering community to invest beyond core algorithms and produce high quality protocol papers that seek to explain what and how a software works while simultaneously providing a user-friendly interface that focuses on productivity (see Haas *et al.* 2013 for an example from a popular RNA-Seq assembly software ^8^). A final point is that genomics – being data-rich - is ideal for exploratory research (i.e. generating new hypotheses). The varying quality of genomes and the inherent noise present in biology can actually be accommodated by approaches residing within the information science field (colloquially known as “big data science”). The information science field has been widely used in other disciplines and there are tangible and immediate benefits in experiments such as those using expression data (c.f. see a perspectives article by Hudson *et al.* 2012 ^9^). The only caveat is that, like all experiments, the quality of any such outcomes will depend on the quality of our resources. In order to avoid surprises, before we proceed with such experiments, we ought to first understand the process of generating such resources.

## Insight 2: A “life cycle” and a grand experiment

**Figure 1.**
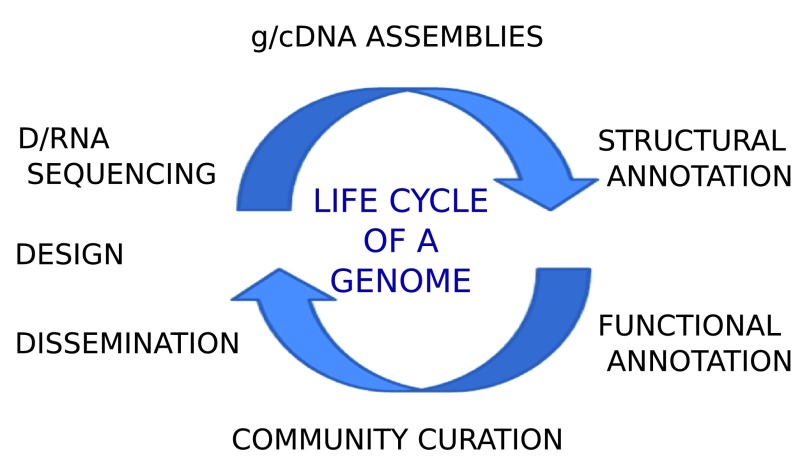
The iterative process of generating a genome sequence can be seen as a life cycle.

From a pedagogical perspective, one can compare genome projects to the life cycle of an insect ( Figure 1). Like a developing insect, genome projects go through several stages of development: project design (often of an underestimated importance); DNA and RNA library preparation and sequencing (with rapidly evolving protocols); genome and transcriptome assembly (initially more than one before a consensus one is decided), structural annotation (e.g. “where are the genes and other features?”); functional annotation (e.g. “what does this gene do?”); manual curation of these two annotation types (often the most time-consuming step); and data dissemination (i.e. the steps that are visible to the public and perhaps the most important stage). Viewing this process as a life cycle provides not only the basis of an improved educational narrative but also some immediate insights. For example, genome project can go through multiple iterations of this “life cycle”. Further, like insects, the fitness of each stage depends on the quality of all of the previous stages. Also notable is the fact that one cannot proceed unless a stage is completed and “frozen” (in sequencing centre jargon, i.e. no longer manipulated). For example, the annotation process cannot begin unless the assembly is completed. Again, this insight may seem intuitive but having it at the forefront of our thoughts while undertaking a genome project will afford us with some important advantages.

Before elaborating on that, it is important to first point out that creating a draft genome sequence is a scientific experiment. There is at least one question (often a biological one, the nature of which depends on the discipline of the research leaders), but at the very least involves investigating an organism’s genetic blueprint. From a computer science point of view the question is straightforward: what is the correct genome sequence for this species and what are the parts that are important for its function. Further, there are a variety of possible methods and approaches that can be used, there is a risk of failure and at the end there will hopefully be more questions than answers. Therefore, like all scientific experiments, a good project design is essential. This ought to be led by someone - or a team - possessing in-depth knowledge of every step of the process. A review by Richards and Murali 2015 ^10^ outlines many of the common issues a team has to consider when working with insect genome projects. For example, DNA availability and quality, genome size and polymorphism are some of the most important aspects that have led to the poor quality of a number of genome projects. As we complete more genome projects, further capturing and sharing that knowledge is something that the community sorely needs.

By perceiving genome projects as an experiment with a life cycle, one can begin identifying a number of useful insights. For the sake of brevity I will expand on only a couple of the most important ones that can help steer genome projects to be more likely to succeed.

First, most genome projects want to address a particular question which varies between disciplines. It may be to perform a quantitative genetic study, fully ascertain a gene family which hosts a number of recently duplicated and near-identical members, or to build a more accurate phylogenetic framework and identify genes that are key innovations. Each one of these aims requires a genome of a different quality and therefore the project design ought to focus on those outcomes. For example, quantitative genetic studies depend on long scaffolds so markers can be associated with causal genotypes. Gene family ascertainment needs not only a high base-level accuracy but also characterisation of any gene family member turnover (i.e. Copy Number Variants) that may exist within a species ^11^. Phylogenomic studies, on the other hand, require neither of these two characteristics: rather multiple species have to be analysed and putative key genes need to be painstakingly curated and characterized. Striving for perfection by achieving all these characteristics could be attempted in the first iteration but rarely do genome project teams have the diversity of skill, time and money to achieve it in a timely fashion.

Second, striving for perfection often manifests as a lack of discipline in keeping with the original experimental design when faced with access to new technological advances. This is even more critical when one considers that the speed of innovation in this field is extraordinary and that new technologies are less well-tested both in terms of wet-lab techniques and the dry-lab algorithms meant to analyse them. For example, even though new approaches such as long read sequencing ^12, 13^, linkage maps ^14, 15^ or chromatin interaction data ^16^ are an under-used approach and can be of great value to improving genome sequence contiguity (i.e. scaffolding), they are high-risk, time consuming and expensive. Unless the original design included them, in practice they will end up delaying genome projects by months if not years. If one accepts the iterative nature of genomes, one can strive for timely incremental improvements.

Third, we ought to remember that the life cycle moves forward only when we are satisfied with the quality and therefore when we are not satisfied we have to backtrack. Most genome sequencing groups learn early on to appreciate the need to complete each stage to a satisfactory level before proceeding to the next stage. Quality assessment using pre-defined metrics is standard practice in data science. More experienced workers also learn that once a stage is satisfactorily completed (“frozen”) and the next one started, one must under no circumstances go back. For example, once the structural annotation is completed, any perturbation of the assembly will invalidate the genome sequence and co-ordinates that gene models depend on. Certainly, if the stage is not satisfactory then it is expected that we go back one step (or even back to stage 1, project design) and start over. For example during the *Helicoverpa* genome project, we found that when there are high levels of polymorphism, the error levels of the 454 technology were prohibiting in completing an assembly at an N50 higher than 30 kb; short Illumina reads (ca. 100 bp at the time) were far more suitable and could be coupled with 454 mate pair libraries to produce a genome assembly of an N50 exceeding 1,000 kb. Had we decided to continue with the life cycle then we would have invested enormous effort in annotating a fragmented genome that was not suitable for our aims.

Fourth, the life cycle enforces the notion that quality of any one step is dependent on the outcomes of the previous step. For example, using the best possible starting material and extensive quality control of the sequencing data can add far more value to an assembly than any wet-lab or even dry-lab investment. This may also appear self-evident but, as mentioned by Richard and Murali, many non-model insect projects have limitations due to biology or availability of samples which no assembly algorithm can account for. For example within the *Heliconius* community, a multi-species genome project undertaken by the Discovar team, showed that even this new approach provides results inferior to the well tested Allpaths-LG approach (created by the same team), even in the hands of the authors. This is postulated to be due to these butterflies exhibiting high levels of complex polymorphism and repeat structures (Owen McMillan pers. comm. September 2015). Instead, a current protocol using a more traditional Allpaths-LG ^17^ strategy coupled with a dense linkage map has allowed for a far superior assembly. At the end of the day, a combined understanding of the computational approaches used in genomics and the genomic architecture of a species is required to generate an excellent assembly; this will likely require more iterations of the life cycle.

## Insight 3: Sharing is caring

Significant resources and team effort are required for updating a genome version. In my experience, the greater of the challenges is how to co-ordinate the release of a new genome so that the community has access to the latest science and the genome team is rewarded for their contribution in way appreciated by their funding bodies and employers. There are not many insect genomes that have completed the life cycle multiple times but a flagship example is that from the silkworm, *Bombyx mori*, which had three publications. The first paper provided an early draft of the genome that turned out to be of limited broad utility but was published in Science ^18^. The second was published shortly afterwards by a second, competing, group ^19^. Even though it was of higher overall accuracy, that paper appeared in the journal of DNA Research (Oxford University Press). The third publication was the result of intense political activity, leadership and labour from both teams. It delivered a genome resource that was of higher quality than any other non-model species published at the time (and for the time being, it still is). However, this last iteration was published in the domain-specific journal of Insect Biochemistry and Molecular Biology ^20^. Further, it is unlikely that any funding body would support any such activity today: once a genome is “published” it is deemed complete. Even though some of the raw data was made available through GenBank, it is important to note that each silkworm paper came with its own data repository and database. Indeed, there is still no “one stop-shop” for silkworm genomics and there is no support for the community to provide feedback for particular scaffolds or genes (i.e. “curation”). By all accounts, even though silkworm genomics is very much alive, it seems that the relevant informatic community is no longer active and I fear that most insect genome projects are – by default rather than choice - following this protocol.

So we are faced with two issues: how to provide informatic support beyond that initial publication and how to create a sustainable publishing model that allows for a genome project life cycle. In this particular case, I believe these two issues have solutions that can address both. One option, currently undertaken by the community, is for the papers exhibiting a new genome version to be submitted as a technical advance to a low impact factor but useful journal. This may, however, prevent engaging the best bioinformaticians and also undervalues the contribution of bioinformatics. Another option is for genome project teams to address a different, novel and important research question. That way the value of an improved genome resource can be properly showcased. This is time-consuming, however, and will therefore result in significant delays for making the data accessible to the community. A third option is to decouple publications from resources while at the same time respecting the value of genomics. We can achieve that if we shifted our focus from the “impact factor route” ( 21; c.f. the San Francisco Declaration on Research Assessment from the American Society for Cell Biology) and focus on “real world impact”. This is what an increasing number of government and research institutions (such as universities) are being asked to focus on. In genomics, we can make use of an updated version of the “Fort Lauderdale Agreement”. First, the data is made available before publication (in a controlled fashion) and the community is offered the opportunity to edit and improve it. Importantly, the community should be able to use it for downstream experiments and - if a journal editor agrees and their work does not fall under genomics but say biochemistry or molecular ecology – publish their findings (e.g. see 22 for an example from the *Helicoverpa* Genome Project). The assembly and annotation are benchmarked by this process and the community acquires both awareness and training. Eventually the first “genome paper” is submitted to a journal. This showcases not a new technical capability or a competition for being “the first but not the best” but rather a broad body of work from a relative large section of the stakeholders. The subsequent genome versions can be linked to either new experimental work on this one species or multi-species comparative genomic insights. Under the leadership of the Baylor College of Medicine, this is exactly the model that the i5k community has chosen. Even though a considerable number of genome projects have been “completed but unpublished”, they are nonetheless available either freely (e.g. the Mediterranean fruit fly is on NCBI) or upon request (for example see https://www.hgsc.bcm.edu/i5k-pilot-project-summary) and there are significant real world impacts as scientists across the world are collaborating using these new resources.

Overall, our community is excellent at producing and disseminating the outcomes of top quality research, however, what the broader community values most is the dissemination and maintenance of data. Except terminal data (e.g. assemblies), primary (e.g. sequencing reads) and annotation data are also extremely valuable for conducting further experiments. A reliable and user-friendly IT platform for dissemination is the most effective way to reduce the bioinformatic bottleneck that is manifesting in many labs. Traditionally, data dissemination occurred in tandem with publication (e.g. via GenBank). Sadly, this is often limited to what occurred to get a particular paper accepted in that one publication and we cannot rely on journal editors to ensure that up-to-date data are available. Informatics has certainly empowered the community by providing it with a number of tools such as those based on “GMOD toolkit” and Content Management Systems ^23, 24^, Ensembl ^25^, InterMine ^26^ or even entire infrastructures that can support the knowledge discovery process from beginning to the end ^27^. Provision of resources is also not limited by a lack of effort (c.f. the Nucleic Acid Research and Database journals) but issues such as lack of funding, maintenance, exchange of data from other resources or communication with the relevant community. As a consequence, their utility or life-span can be limited and the invested informatic effort wasted. We need a system that has the interoperability of the UCSC Browser (the version developed for cancer research; 28), the web-services of InterMine (created originally for Drosophila; 26), the data richness of the ENSEMBL project ^25^ and the ecosystem of iPlant ^27^. At the same time it hosts a dedicated team knowledgeable on insect biology and tasked with not only managing the data for the insect community but also building awareness for best practices, providing training and enforcing quality control. Without this resource, every new insect genome that is funded will be of limited value. The major issue for ensuring long-term sustainability is that sequencing centres and science leaders cannot guarantee the long-term provision of the required computational infrastructure. Even though centres such as the NCBI can host raw data and finalised gene models, they cannot provide a community portal with domain specific tools and resources. This is another area where the i5k consortium has shown leadership: in collaboration with the National Agricultural Library (NAL) of the USDA the insect community has now access to a dedicated team which is deploying an increasing number of tools (including the GMOD toolkit) and provides basic computational resources and training ^29^.

## Insight 4: The human touch

The NAL team goes beyond merely hosting data and developing tools: they provide a platform for the community to assess the quality of genomes and edit the results of the automated bioinformatic processes of annotation. This manual checking and editing, i.e. “curating”, is an important check on the automated approaches on the underlying data that any experiment will end up relying on and it has featured in all major genome projects. In the early days of genome projects, the automated annotation ‘freeze’ was the stage where significant community outreach and involvement was sought. This often took the form of Annotation Jamborees and these were driven – and funded - by the leaders of the consortium. There, community members would meet and edit the computational predictions using the Apollo annotation system ^30^, discuss research questions and co-ordinate project activities. These events are now mostly associated with the Sanger era where the costs to create a genome sequence were orders of magnitude larger than the costs associated with hosting a meeting. However, these meetings played a critical role in not only improving on the computational predictions but also forming a genomics community and educating researchers on how to use the genome ^31^. As genome project costs have been driven down we had to invent new ways of co-ordinating work. One solution has been the International Arthropod Genomics Workshop but that lacks the immediacy and cannot deal with the enormous volume of data and diversity of species in a timely manner. Perhaps not surprising, informatics came to the rescue with a number of ‘community curation’ tools developed. In the insect world, the clear winner has been the Web Apollo software (also known as WebApollo) a plugin of the JBrowse genome browser ^32, 33^. Except for offering a real-time, internet-enabled implementation of genome viewing and editing, this informatic capability is underpinning NAL’s effort to help in forming, educating and maintaining genomic communities. Our greatest challenge in this space, however, is that we are one step behind: even though we are excellent in collecting and curating genomic data, we will still have to learn how to efficiently collect and curate a vast amount of new types of information such as those derived from epigenetics, population genetics and even ecology.

## Future directions

If there was one final take home message it would be that while the genomics community is currently reaping the benefits of a number of technological advances, it is also about to be faced with a paradigm shift due to not only the number of genome sequences being made available but also the types of data that are becoming cheaper and increasingly common. Certainly, we need more scientists to learn how these data are derived and how to work with them more effectively but we – the informatics community - also need to educate more of them of how to produce high-quality, long-living projects that meet best practice. In my opinion, the challenge to deliver such an outreach activity is the development and the delivery of a high quality, unified course that will perhaps be tailored for each taxonomic domain or discipline. It is true that genome analysis and bioinformatics is a research discipline that takes years to master but – like statistics – it is also a useful set of tools that empowers everyone who chooses to invest the time to acquire some basic knowledge. Even further, it is such an exciting time to be a biological data scientist that those who decide to view computational biology as a skill to be mastered, while excelling in their chosen biological discipline, may also drive many of the next generation of synthesis in biology.
