# Supplementary material for: The life cycle of a genome project: perspectives and guidelines inspired by insect genome projects
Source: F1000Res. 2016 Jan 5;5:18. [Version 1] doi: 10.12688/f1000research.7559.1 (PMC4798206; doi:10.12688/f1000research.7559.1)
Supplement: Peer review history

# Peer review: Referee response for version 1

**DOI:** 10.5256/f1000research.8139.r11817

**Published:** 2016-03-15

**Authors:** Ian Holmes

**Affiliations:** 1 Department of Bioengineering, University of California Berkeley, Berkeley, CA, USA

This is a very nice opinion piece surveying the current community practices around genome projects and the shortcomings thereof. Using the metaphor of the insect lifecycle, the paper discusses the “genome lifecycle” (sequencing, assembly, annotation… re-sequencing, re-annotation, etc) and various lessons drawn from real-life case studies (e.g. informatics is key, perfection is the enemy of the good, we need to decouple data dissemination from publication to some extent, we need plans for sustained computational infrastructure, we need new collaborative tools).

I agree with the positions espoused here and I find this piece a very insightful distillation of the challenges facing the community as funding pay-lines become tighter and we transition from an era of quick genome-project headlines to one in which the community can (with luck) collectively curate and maintain data, rather than letting data-silos decay.

I had one minor suggested edit which is that the line “until the skill and leadership of Jim Kent saved the day” could use a citation (presumably to Kent & Haussler, 2001 ^1^).

I have read this submission. I believe that I have an appropriate level of expertise to confirm that it is of an acceptable scientific standard.

## References

[1] KentWJHausslerD: Assembly of the working draft of the human genome with GigAssembler. *Genome Res*.2001;11(9) : 10.1101/gr.183201 1541-8 10.1101/gr.183201 11544197PMC311095

# Peer review: Referee response for version 1

**DOI:** 10.5256/f1000research.8139.r11819

**Published:** 2016-01-15

**Authors:** John W. Davey

**Affiliations:** 1 Department of Zoology, University of Cambridge, Cambridge, UK

This article draws attention to several important issues related to the production and use of reference genomes and associated data sets, particularly gene annotations. It outlines the typical process for a genome project in the era when large consortia could acquire substantial funding for such a project and produce high-impact genome papers. It correctly notes that the process of generating genomes is rapidly changing, as sequencing costs are falling and tools are improving, allowing small groups to produce genomes for a fraction of previous costs, but also with reduced impact. This change has many scientific and political implications for the production of genomes, which the article attempts to summarise. However, I do not think it does a good job of summarising or addressing these implications. I hope the following criticisms will help to bring these important issues into the clearest possible light.

The article highlights the conflict between the nature of a reference genome as a resource that requires long-term, communal effort and infrastructure, and the nature of science funding, which requires the repeated completion of substantial short-term goals leading to high-impact first- and last-author papers. It claims that genome projects are best undertaken with particular biological questions in mind, in order to deliver the most relevant possible resource at the time and to avoid perfectionism and the distraction of new sequencing technologies and assembly tools. It also rightly insists that genome projects require complex computational analyses that need to be understood to some extent by those producing and using the genome so that potential errors in analyses using the genome are well understood and can be fixed where possible. It calls for the active building and maintenance of communities of researchers working on particular genomes, partly through the training of scientists in genome assembly and annotation techniques. It also calls for the decoupling of publications from resources by rewarding efforts for their real world impacts, and for increasing the likelihood of real world impacts by making genomes available early and encouraging communities of researchers to use them. Finally, it highlights the importance of core teams such as that at the National Agricultural Library for disseminating training, hosting data, and providing tools and platforms in order to support individual groups working on genome projects, and the need to secure long-term funding for these teams.

All of these points are important and worth making strongly (with a few caveats), both for those already involved in genome assembly and annotation and those new to the field. But they do not come across clearly in the article, as they are hobbled by inconsistent use of various concepts and by several bad examples that hurt the case being made.

Firstly, the concept of a community is very unclear. There are 44 references to communities in the article, including the non-model species community, the genomics community, the insect community, the insect genomics community, the research community, the wider community, the software engineering community, the i5k community and the informatics community, but mostly just to 'the community'. There are then 39 references to what 'we' are doing or should do. Who are we? Which community or communities does 'we' refer to in each case, and who is the article addressed to? It is not clear who needs to do what differently in order to improve the situation, or where the problems really lie. To give one example, "Third, the research community is not just the end-user but also part of the project team; we have, on the whole, neglected to bring them up to speed. These issues may seem intuitive but much of the community’s leadership is not conscious of it." Who have neglected to bring the research community up to speed? i5k? Informaticians? Which community's leadership is not conscious of the problems, the i5k community, the informatics community, the research community? The article at points seems to be attempting to address a general audience, but other times is directed to the i5k community, and at points seems to be saying i5k should be doing things differently, but at others recommending the i5k model as best practice. While these things are not necessarily incompatible, the article would be much easier to read if it was much clearer about the groups it is addressing, and the social structures that would improve the process of generating genomes.

Secondly, the contrast between using genomes to answer questions and providing genomes as resources is passed over, with both being claimed as important while not addressing the conflict between them. 'Genomics' is sometimes used to refer strictly to the production of genome sequences and perhaps annotations, but sometimes to research done using these sequences and annotations. The concept of a genome project being an experiment is confused in the same way; sometimes it seems to refer to the genome assembly as an experiment itself, and sometimes to the genome as used to conduct an experiment that answers a biological question (which can drive the genome project design). And the same confusion arises over the encouragement for scientists to learn 'data science' and related fields; sometimes this is directly related to genome assembly, sometimes to research using the genome.

Clarity on these issues is important, because at present the confusion obscures some hard problems. For example, while it is highly desirable to direct genome projects towards particular biological questions (to maximise the chance of funding and high-profile papers, and to circumscribe the limits of the genome project itself), and to engage the widest possible relevant community in the production of genome resources (to make sure the genomes are used correctly, to increase impact, and hopefully to increase quality of assembly and annotation), the article doesn't bring out explicitly the fact that these goals are antithetical. As more groups become involved in a genome project, the number of relevant biological questions increases, and the quality of the genome must increase to accommodate them, making it harder to design and manage the project (especially if the entire community is to be involved in not only the annotation but also the assembly, and do research on the genome along the way, as recommended in Insight 3).

Also, while the article makes several welcome calls for better genomics education, the confusions over what the relevant communities are and the distinction between use and provision of genomes make it very unclear what the nature and extent of this education should be. Is the article arguing that bioinformaticians should do the assemblies but educate biologists in the limitations of genomes they deliver; or that bioinformaticians should train biologists to assemble and annotate genomes themselves; or that the role of bioinformatician should disappear and biologists should do it all themselves, given that all biology is computational these days; or that biologists should use more data science techniques on their research but leave the genome assembly up to dedicated bioinformaticians? The article seems to be arguing for variations on these possibilities at different points.

I don't know what the answers are to these problems, but at least they should be brought out clearly in the article, rather than left obscure. With this in mind, I will turn to individual comments on the insight sections.

Insight 1: there is an important point here, which is that results may vary greatly depending on how software is used, and a more basic one, which is that computation is (and always has been) required to produce genomes and so those who wish to produce a genome need to engage with computational analyses. But these points are obscured by more confusions and irrelevant or inaccurate points.

The opening point, that genomics has moved from being led by and limited by wet lab techniques to being led by computational science, is highly debatable, and I personally don't agree, unless perhaps if 'genomics' here means biology in general. Genomics in the sense of genome assembly continues to be led by the available sequencing technologies, not by computation - the two current assembly methods referred to in the paper, Allpaths and Discovar, were both designed to fit an available sequencing technology, they did not prompt the development of the technology. In long read sequencing too, the technology is driving the algorithms, not the other way around. While the Celera assembler is a great achievement and is being heavily used to assemble long read sequences, it is far from the case that Pacific Biosciences and Oxford Nanopore are designing their machines to fit the design of the Celera assembler. And the human genome example doesn't support the case at all, given that, as noted, 'the WGS approach [was] rightly considered to be of inferior quality' and the private genome ended up incorporating a lot of the public mapping data; the article ends up concluding that it was the 'cross-talk of the two capabilities' that was important, contradicting the initial point of the paragraph.

The second paragraph attempts to make the case for biologists to develop computational skills, but the range of terms used just further obfuscates the issue. What is the difference between information technologies, informatics, data science, information science and "big data science"? How exactly are they related to genomics and bioinformatics? What distinct 'epistemological understanding' does statistics and informatics provide that biology does not, and what is a 'framework to make sense of a more synthesized knowledge' (and why should a researcher want it)? If the point is to say biologists would benefit in general if they improved their computational skills, that may be true, but is isn't really relevant to an article about genome assembly (and it is mildly insulting to say biologists need to improve their statistical skills, given that they invented statistics). If the point is to say biologists need to engage with genome assembly and annotation, that's quite a different issue, and doesn't need to be backed up by the general case for computational training. Reducing the generalities about computation and increasing the specificities about how biologists need to engage with genome assembly and annotation would help here.

Insight 2: again, several very different points are mixed up into one here. Genome projects to date do tend to follow a life cycle as described, and can be iterated. But the points that follow, especially those about experiments, are confused. It is true that a genome sequence can be used to test hypotheses, and that the relevant hypotheses can often direct the design of a genome project. But in what sense is 'creating a draft genome sequence' an experiment? What is the hypothesis being tested by the assembly process itself? In what sense is 'investigating an organism's genetic blueprint' a hypothesis-driven experiment? It's possible to make the analogy (perhaps every time an assembler compares two reads, it conducts an experiment to test whether the reads overlap or not?) but it is not very enlightening, and it is not necessary for making the case that good project design is essential, that a variety of methods can be used and that there is a risk of failure - many things other than experiments share these properties. The sentence about the computer science point of view is even more confusing; the question "what is the correct genome sequence for this species" does not require an experiment in the traditional sense, and "what are the parts that are important for its function" isn't really a computation-only question at all. 

Further, the advice here is quite convoluted - "when we are not satisfied we have to backtrack", but "More experienced workers also learn that once a stage is satisfactorily completed... one must under no circumstances go back", however, "if the stage is not satisfactory that... we go back one step". Clearly satisfaction is key here, but our satisfaction can change - and if our satisfaction about an earlier stage is changed by what we discover at a later stage, does that mean "one must under no circumstances go back"?

While genome projects to date have followed a life cycle as described, and perhaps initial versions of a genome may need to follow this process, I'm not convinced that a strict adherence to this model for future iterations is helpful. Insisting that every stage of the life cycle is completed by the whole community step by step in order to lead to a paper of lower and lower impact is surely the model we want to get away from. There is decades of research in software engineering refining or rejecting completely this kind of waterfall model in favour of more incremental approaches; while there is still controversy over this, it seems likely that genomics could benefit from moving in this direction as well.

In theory, there is no reason why genomes can't be patched and updated piecemeal as small assembly errors are fixed, or scaffolds are ordered, or single gene families are annotated, with infrequent major releases rolling together these patches. This is standard practice in the software industry and for the human genome. I don't claim this is the only way to do things, or that there aren't problems with this approach, and it is true the infrastructure is not in place to do this efficiently for non-model species. But that doesn't mean we should restrict ourselves to the existing life cycle model; adhering to this model is one of the causes of the problems the article is trying to address (big version releases lead to problems in acquiring funding, managing large communities, rewarding individual contributors, deciding on publication strategy...). Why not just change the model?

Finally, the point about genome assembly often being limited by the biology of the organism is valid, but the example is a poor fit and should be removed. The Heliconius Discovar assemblies were never intended to provide reference-quality assemblies, as the Allpaths-LG assembly was, and the biology of the organism was not ignored, as the paragraph implies; in fact, the Discovar assemblies were specifically intended to test the Discovar assembler on a set of highly heterozygous genomes, and improve the assembler to deal with this data. The assemblies were preliminary and were never optimised because the Discovar team left the Broad and did not complete the project, so it isn't fair to compare the assemblies. A better example to support this point would be the Plutella xylostella genome, where considerable heterozygosity remained after ten generations of inbreeding and thorough fosmid sequencing was required to produce a genome of reasonable quality.

Insight 3: the issues described here (how to provide informatic support beyond the initial publication and how to create a sustainable publishing model that allows for a genome project life cycle) are real, but the solutions provided are not very realistic, are already fairly standard practice, or do not address the issues. Three options are presented: submit new genome versions as low-impact technical papers; use the new version to address a new biological question, or (the preferred option) to decouple publications from resources and respect the value of the genomic resources. This last option might well be a good idea, but the proposals for achieving it fall short.

Most of the points made (releasing data early, engaging the community and allowing them to publish before the genome is published in its own right, showcasing a wide variety of analyses in the eventual genome paper) are to do with the initial release of the genome, not how to maintain the genome beyond its initial publication. There isn't much new in these points, given that this is the template set by the human genome project, but that doesn't necessarily mean it's not worth highlighting them again. However, it should be noted that this very fluid use of data, where the community edits and improves the assembly and annotation, makes maintaining a strict life cycle with frozen stages even harder.

The only point this paragraph does make about later versions of the genome is that they should be linked to new experimental work or multi-species comparative genomic insights - which is just the second option that was passed over earlier. Also, the first option is passed over because it is unappealing to the best bioinformaticians, but why should a bioinformatician working under the standard publishing model where first-author papers are required be more interested in the proposed model where a large range of community analyses, some perhaps previously published (and so lowering their impact or making them inadmissable for further publication), are put into one paper?

The problem is correctly identified as the conflict between the publishing model for individual scientists and the need to build communal resources, but the text doesn't propose anything meaningful to address this, beyond insisting that it would be good to separate publications from resources. But how is this to be done? Which communities need to change what they are doing, and how they value work, to achieve this? What metrics should we be using and recommending to faculty in hiring computational biologists, other than publications? While touching on this issue, the article does not really address it, and does not extended 'real world impact' beyond the use of the data by other researchers. If this is the limit, what is wrong with the current system where impact is measured by the proxy of citations?

Finally, the whole manuscript would benefit from more attention to detail. For example, "Second, this draft does indeed contain many of the instructions of how to generate an organism, but a genome sequence alone does not decipher it. It merely transcribes so we can conduct experiments with it. Deciphering will require both good experimental design and the capability to integrate such experiments." - what do the two consecutive 'it's refer to? The organism, then the genome sequence? How does a genome sequence transcribe? What is being deciphered? What are the experiments being integrated with? "this number is increasing exponentially" - is it exponential? "an achievement which may be currently underutilised but whose importance cannot be understated" - surely overstated, but the hyperbole doesn't help here anyway. It's not convincing to just say the work is important; why is it so important?

I am sorry to be so critical, especially in public. I hope this level of detail will be taken as a mark of respect for Dr Papanicolaou's expertise and passion for this subject, which I agree is a very important topic that needs to be engaged with by all involved. I thank him for stepping forward to raise these issues and hope that this review will be taken constructively and lead to improvements in the piece.

The following typos or omitted words should be fixed:

the genomes projects of a larger part the tree of life

One the major forces of innovation

dataset: The

explain what and how a software works 

For example, genome project can go through

allowed us generate genomes

Richard and Murali

for their contribution in way appreciated by

on the automated approaches on the underlying data 

that lacks the immediacy and 

Except for offering a real-time -> Because?

may also drive many of the next generation of synthesis in biology. -> syntheses?

I have read this submission. I believe that I have an appropriate level of expertise to confirm that it is of an acceptable scientific standard, however I have significant reservations, as outlined above.

# Peer review: Referee response for version 1

**DOI:** 10.5256/f1000research.8139.r11905

**Published:** 2016-01-12

**Authors:** Stephen Richards

**Affiliations:** 1 Department of Molecular and Human Genetics, Baylor College of Medicine, Houston, TX, USA

This is an excellent review of the genome project process and life cycle, that most valuably shows the reader how a genome project fits into the larger goal of biological research around a species. Dr. Papanicolaou's insights remind us how genome scale data "completely changed our perception of how biological research can scale in a world that transcends borders" and provides passionate enthusiasm and advice for those researchers and communities without genomes who wish to join this new world.

Insight 1 tells us about the power and necessity of bioinformatics for not just assembly and annotation, but the genome wide analyses required to gain the most biological insight, but additionally warns us against relying on the "black box" that software can become without understanding how it works. I for one have fallen for this by following the conventional wisdom about what a particular software package does, but finding out only by inspection of the code that something else entirely is going on.

Insight 2 places the genome project in it's rightful place as part of the experimental life cycle. The emphasis on experimental design in genomics is important - all to often in the past this critical step is ignored either due to cost reasons in collecting a sufficient data set compared with the urge to do something and call it preliminary data or simply out of bravado and not planning, with the result of very poor genome assembly quality and unreliable or un-interpretable downstream analyses. Dr. Papanicolaou outlines the importance of things like community curation to simply enable the community to look closely at the data and gene models - something that is vital to get an idea of how much to trust any conclusions coming out. but at the same time to match genome quality to the desired experimental requirements, to freeze genomes and annotations, and to get on with it and publish. This is excellent advice, and many a genome projects publication has been stalled for multiple years in the pursuit of better quality without the contaminant investment of resources, or in slow transition between the various steps of analysis dues to poor planning and training for the entire process of the life cycle. This part of the review is required reading for anyone contemplating a genome project, directing the thoughts of the reader to consider the longer term plan for the genome for his or her's lab, experiment or even for a larger community, and tailoring the experimental plan to fit.

The insights on Data Sharing, and the requirement for pre-publication data sharing are critical, and point the reader to resources that will enable placing new genome datasets in public repositories with long funding horizons, stable futures, and academic reach around the board. More interesting to this reviewer, was the discussion on the difficulties in funding and publishing the improvement of draft genomes in the future. Although this is getting technically easier with he advent of longer read sequencing technologies, the manuscript is correct in noting the difficulties in publishing a fourth improved draft genome compared with the third - it is hard to say it is a significant improvement to our state of knowledge when closing say 75% of the gaps. I believe in the future we will still be interested in "effectively finished" archival genomes, and that these will be worth data notes in lower impact journals, but the option of "decouple publications from resources while at the same time respecting the value of genomics " to me seems like the correct way forward as we one day hope to have sequenced all species on the planet - i.e. to read the primary biological data for life on earth. Whilst we realize the genome sequence of the 10,000th bird species may not make the highest profile journal, not to have this sequence in the natural history museums of the future seems unthinkable.

The human touch insight is dedicated to the need for researchers to look at data to correct gene models, to understand the limits of the dataset. New tools allow this to be done in a co-ordinated manner with groups of researchers from around the world, with the result that research can be accelerated around the world with the sharing of a single genome. This is particularly true today with the use of RNAi and Crispr gene manipulation techniques. In the milkweed bug community RNAi was the mainstay of comparative developmental research, but relied on degenerate PCR to identify genes and design probes. A draft genome quickly gave this research community the information to design all the probes they needed, but human curation was still needed to checkoff the number of genes in a family had changed from the Drosophila model, or that the automated gene model had got the sequence right before committing to a wet lab experiment, and that phylogenetic trees had confirmed that the researcher was manipulating the gene he or she thought she was, and not a paralog or a gene from a different but related family.

Overall Dr. Papanicolaou has written an excellent guide to the genome project, the reading of which will profit anyone contemplating a genome project. It is well written, and whilst I have a few differences of opinion on minor points, they are in no means enough to prevent indexation. Overall I believe this manuscript merits immediate indexation with no modification necessary.

Bonus points for remembering and reminding us of the role of Jim Kent.

I have read this submission. I believe that I have an appropriate level of expertise to confirm that it is of an acceptable scientific standard.
